# Implementing a combined infection prevention and control with antimicrobial stewardship joint program to prevent caesarean section surgical site infections and antimicrobial resistance: a Tanzanian tertiary hospital experience

**DOI:** 10.1186/s13756-020-00740-7

**Published:** 2020-05-19

**Authors:** Elisa Gentilotti, Pasquale De Nardo, Boniface Nguhuni, Alessandro Piscini, Caroline Damian, Francesco Vairo, Zainab Chaula, Paola Mencarini, Peter Torokaa, Alimuddin Zumla, Emanuele Nicastri, Giuseppe Ippolito

**Affiliations:** 1 ”Lazzaro Spallanzani” National Institute for Infectious Diseases-IRCCS, Via Portuense 292, Rome, Italy; 2 Resource Centre for Infectious Diseases, Dodoma Regional Referral Hospital, Dodoma, Tanzania; 3 Gynaecology and Obstetrics Department, Dodoma Regional Referral Hospital, Dodoma, Tanzania; 4 Division of Infection and Immunity, Centre for Clinical Microbiology, University College London, London, UK; 5 National Institute of Health Research Biomedical, Research Centre at UCL Hospitals, London, UK

**Keywords:** Caesarean section, Surgical site infection, Antimicrobial resistance, Antimicrobial stewardship, Resource-limited settings

## Abstract

**Background:**

Surgical site infections are a leading cause of morbidity and mortality after caesarean section, especially in Low and Middle Income Countries. We hypothesized that a combined infection prevention and control with antimicrobial stewardship joint program would decrease the rate of post- caesarean section surgical site infections at the Obstetrics & Gynaecology Department of a Tanzanian tertiary hospital.

**Methods:**

The intervention included: 1. formal and on-job trainings on infection prevention and control; 2. evidence-based education on antimicrobial resistance and good antimicrobial prescribing practice. A second survey was performed to determine the impact of the intervention. The primary outcome of the study was post-caesarean section surgical site infections prevalence and secondary outcome the determinant factors of surgical site infections before/after the intervention and overall. The microbiological characteristics and patterns of antimicrobial resistance were ascertained.

**Results:**

Total 464 and 573 women were surveyed before and after the intervention, respectively. After the intervention, the antibiotic prophylaxis was administered to a significantly higher number of patients (98% vs 2%, *p* < 0.001), caesarean sections were performed by more qualified operators (40% vs 28%, *p* = 0.001), with higher rates of *Pfannenstiel* skin incisions (29% vs 18%, *p* < 0.001) and of absorbable continuous intradermic sutures (30% vs 19%, *p* < 0.001). The total number of post-caesarean section surgical site infections was 225 (48%) in the pre-intervention and 95 (17%) in the post intervention group (*p* < 0.001). A low prevalence of gram-positive isolates and of methicillin-resistant *Staphylococus aureus* was detected in the post-intervention survey.

**Conclusions:**

Further researches are needed to better understand the potential of a hospital-based multidisciplinary approach to surgical site infections and antimicrobial resistance prevention in resource-constrained settings.

## Background

Health-care associated infections (HAIs) and antimicrobial resistance (AMR) are major global health challenges recognized worldwide The spread of HAIs and AMR is particularly alarming in low and middle income countries (LMICs) [1, 2] where high level of resistance to commonly prescribed antibiotics together with lack of local AMR surveillance systems need urgent attention [3, 4]. According to the World Health Organization (WHO), HAIs are increasing at alarming high rates in LMICs, being two- to 20-times higher than in high-income countries [5].

Surgical Site infection (SSI) is the most frequent cause of HAIs in LMICs, affecting up to one third of patients undergoing surgery [6]. In light of this, WHO guidelines for SSI prevention outlined a roadmap for quality improvement and suggested a multimodal strategy and multidisciplinary approach [7–9]. A cultural and attitude change is essential to ensure that protocols for Infection Prevention and Control (IPC) and Antimicrobial Stewardship (AMS) are strictly followed [10]. Although SSIs are largely avoidable, in LMICs they continue to be a leading cause of morbidity and mortality also among women undergoing caesarean section (CS), a live-saving procedure classified as a clean-contaminated operative wound according to Centre for Diseases Control (CDC) Classification [11].

Dodoma Regional Referral Hospital (DRRH) is one of the few centres of the Dodoma Region, Tanzania, offering CS services. A prospective observational study conducted in 2013 at the Obstetrics & Gynaecology Department (OGD) of DRRH reported an extremely high prevalence of CS-SSIs (48.2%), 40.6% of them caused by *Staphylococcus aureus.* Seventy-nine percent of the *Staphylococcus aureus* detected were methicillin-resistant (MRSA) [12]. A recent before-after intervention cohort study, conducted in five hospitals of sub-Saharan Africa, found that a multimodal SSI prevention strategy in low-resource settings can reduce the risk of SSIs [13].

Our study aimed to assess the impact of the implementation of a combined IPC with AMS joint program on the prevalence of CS associated SSIs at DRRH.

## Methods

### Study design

A before-after intervention cohort study was conducted during 2 years starting from August 2013 at the OGD of DRRH. The study included a pre-intervention survey (PRE-Int), an intervention and a post-intervention survey (POST-Int). Each of the two surveys lasted 3 months and were conducted during the dry season. The study was directly supported by the Resource Centre for Infectious Diseases (RCID) of DRRH. The intervention included literature-based education, on-job training and the constitution of an AMS multidisciplinary team.

### Study sites and study population

DRRH has a bed capacity of 580 and 5 operating theatres, one of them dedicated to the OGD. Data available from 2013 to 2015 report an average number of 14,800 deliveries per year, with approximately 2700 (18%) CS.

### Study procedures

**Fig. 1:**
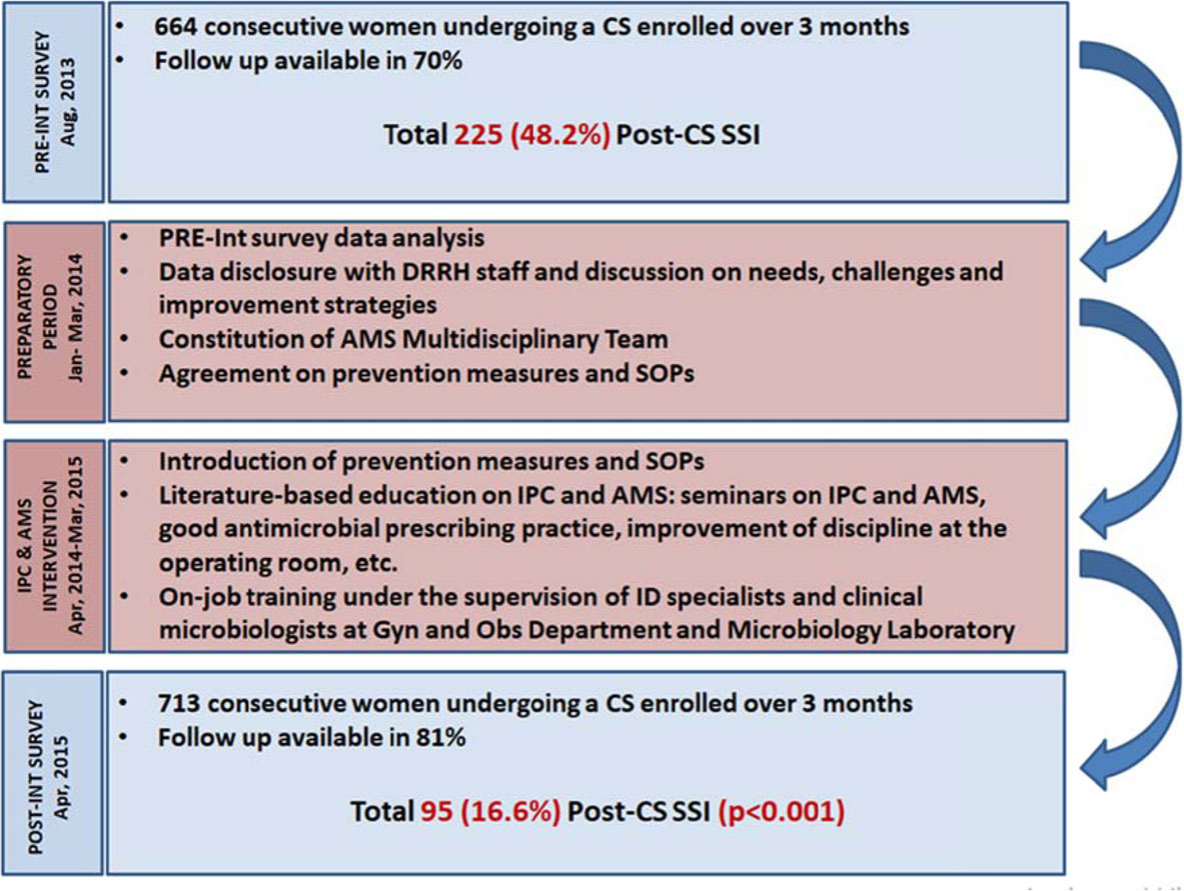
Four-steps protocol of the study. PRE-Int = pre-intervention; CS = caesarean section; SSI=Surgical Site Infections; DRRH = Dodoma Regional Referral Hospital; AMS = Antimicrobial stewardship; SOPs = Standard Operating Procedures; IPC=Infection Prevention and Control; ID = Infectious Diseases; OGD = Obstetrics & Gynaecology Department; POST-Int = post-intervention

A four-steps protocol was adopted (Fig. 1). The first step consisted of the PRE-Int, enrolling all consecutive women undergoing a CS during 3 months from August 19, 2013, with a 30 days post-CS follow up. During the second step, data from the PRE-Int were shared with the hospital staff. Therefore, an AMS Multidisciplinary Team was constituted, including an Infectious Diseases (ID) Specialist, the head of OGD, a pharmacist, an IPC nurse, a clinical microbiologist and a representative of the hospital management. These professionals identified the prevention measures and the interventions to be prioritized: a) implementing the reporting system; b) strengthening the supply chain for antibiotics, disinfectants and operating room/laboratory disposables; c) proper surgical hand preparation; d) administration of a pre-operative prophylaxis 30–60 min before the incision; e) optimizing the appropriateness of antibiotic prescription in the post-operative period; f) improving operating theatre discipline and organization and g) strengthening the capability and capacity of the Microbiology Unit. In particular, the importance of the prophylaxis was stressed. In fact, before the intervention, almost every woman received an antibiotic course lasting 8–10 days post-CS, irrespective of the presence of risk factors or signs/symptoms of infection. The antibiotic course usually included 3 days of intravenous ceftriaxone plus metronidazole, followed by oral penicillin (amoxicillin alone or ampicillin/cloxacillin) plus metronidazole for at least 5 days. The timing was highly variable, ranging from 1 to 24 h post-CS. A pre-operative prophylaxis with ampicillin 1 g given 30–60 min before the incision was suggested, based on drugs availability. The third step consisted in the introduction of the prevention measures into clinical practice and in the organization of seminars focusing on IPC and AMS. Literature-based education was encouraged. On-job trainings were also conducted under the supervision of a pool of ID specialists and clinical microbiologists. Standard Operating Procedures (SOPs), recent publications, guidelines, expert consultation and mentorship on data collection were available at the RCID from Monday to Friday. The last step included the POST-Int, enrolling all consecutive women undergoing a CS during 3 months starting from April 1, 2015, followed by 30 days post-CS surveillance.

### Survey and laboratory procedures

A 30-days follow up was conducted, including a variable number of visits starting from day 7 post-CS. We aimed to have at least one post-operative contact with the patient, either by clinical visit or telephone [14]. Patients were considered lost to follow up after five unsuccessful attempts by telephone. At each visit, an examination of the wound was performed by an ID specialist and the antibiotic treatment history was collected. In case of telephone contact, a structured interview was used to detect SSIs. In the suspicion of SSI, the patient was referred to the nearest health centre. The classification of SSIs was done according to CDC definitions [11]. A wound swab was collected in any case of suspicion of SSI. The specimens were processed soon after collection. Briefly, the specimens were inoculated on blood agar and MacConkey agar and incubated aerobically. Petri dishes were checked after 24 and 48 h for bacteria detection and identification. Antimicrobial susceptibility of isolates was determined using disc diffusion method. The antibiotics tested included: oxacillin (1 μg), ampicillin (10 μg), amoxicillin (25 μg), amoxicillin/clavulanate (20 μg + 10 μg), clindamycin (2 μg), erythromycin (15 μg), ciprofloxacin (5 μg), trimethoprim-sulfamethoxazole (1.25 μg + 23.75 μg), ceftriaxone (30 μg), chloramphenicol (30 μg), gentamicin (10 μg), tetracycline (30 μg). Vancomycin 5 μg, ceftazidime 10 μg and meropenem 10 μg were also tested in the POST-Int study. Based on Kirby-Bauer susceptibility test, gram-negative bacteria were classified as multidrug resistant organisms (MDROs) if resistances to amoxicillin/clavulanate, ceftriaxone (or ceftazidime), and/or gentamycin, and/or ciprofloxacin were detected. MRSA were identified by using the diffusion method with oxacillin disc [15, 16].

### Outcomes

The primary outcome was to report the CS-SSIs rate. The secondary outcome was to assess the determinant factors of SSIs before/after the intervention and overall. The microbiological characteristics and patterns of AMR were determined to provide an overall picture of the SSIs.

### Data collection and statistical analysis

All consecutive CS performed during the study period were eligible for inclusion in the analysis. Data were retrieved from different sources, including: hospital medical records, antenatal cards, surgical notes and structured telephone interviews questionnaires. Data collection was done by trained staff from the RCID and entered in a dedicated Microsoft Excel dataset. Comparison of mean values was done using the Student’s *t* test. The χ^2^ test and Fisher’s exact test were used to explore univariate associations between categorical variables. Log binomial regression model was adopted for multivariate analysis to detect the association between predictor variables and SSIs and to assess the impact of the intervention on outcomes. Co-variates with *p* < 0.1 and considered to be relevant based on clinical knowledge and available evidences, were included in the multivariate analysis. Odd ratios and confidence intervals were computed. A *p* < 0.05 was considered significant. The statistical analysis was performed using SPSS (software version 21, NY, U.S.A.).

## Results

### Comparison of demographic and surgical procedure characteristics

**Fig. 2:**
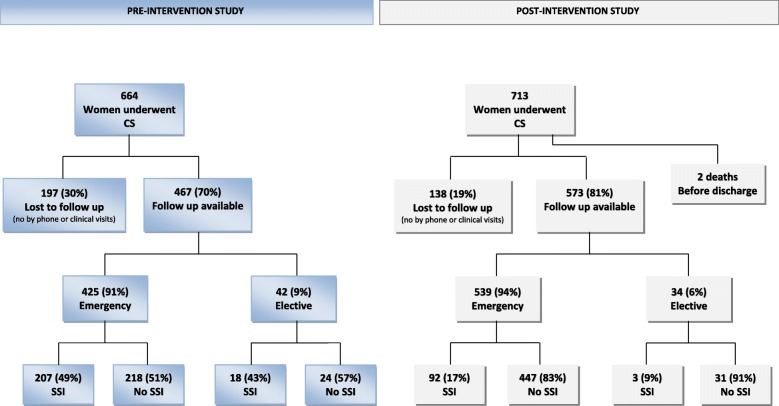
Number of patients surveyed for post-CS SSI in the Pre-Intervention and Post-Intervention groups

Overall, 1377 women with CS were enrolled, 664 (48.2%) in the PRE-Int study and 713 (51.8%) in the POST-Int study. The follow-up was available for 1040 women, 467 (70%) and 573 (81%) in the two studies, respectively (Fig. 2). A total number of 337 (25%) women (197 and 140 in the two studies, respectively) were lost to follow up. The demographic characteristics were similar in the two populations. In the POST-Int a higher proportion of women had a college/university education (adj. *p* = 0.027), but SSIs prevalence was not affected by the level of education.

Table 1 presents the CS characteristics and the surgical procedures in all enrolled patients with available follow-up and in the two study populations, separately (Table 1).

**Table 1:** Overall and per-groups analysis of surgical procedure and CS-SSI Characteristics

	Overall (*n* = 1040) n (col%)	PRE-Int (*n* = 467) n (col%)	POST-Int (*n* = 573) n (col%)	*p*-value	adj-*p*-value*
**CS type**				0.059	0.170^β^
Elective	76 (7.3)	42 (9.0)	34 (5.9)		
Emergency	964 (92.7)	425 (91.0)	539 (94.1)		
**Reason for CS**				0.043	0.155^γ^
CS indication	431 (41.4)	209 (44.8)	222 (38.7)		
No CS indication	606 (58.3)	255 (54.6)	351 (61.3)		
Not known	3 (0.3)	3 (0.6)	0		
**Operator grade**				0.001	0.052 ^γ^
Senior	345 (33.1)	127 (27.2)	218 (38.1)		
Junior	685 (65.9)	337 (72.2)	348 60.7)		
Not known	10 (1.0)	3 (0.6)	7 (1.2)		
**Skin disinfection**				0.000	0.001 ^γ^
Dettol	2 (0.2)	0	2 (0.3)		
Ethanol	94 (9.0)	90 (19.3)	4 (0.7)		
Povidone/Iodine	923 (88.8)	366 (78.4)	557 (97.2)		
Not known	21 (2.0)	11 (2.4)	10 (1.7)		
**Tipe of CS incision**				0.000	0.058 ^γ^
*Pfannenstiel*	247 (23.8)	82 (17.6)	165 (28.8)		
Midline Vertical	737 (70.9)	341 (73.0)	396 (69.1)		
Not known	56 (5.4)	44 (9.4)	12 (2.1)		
**Material used for skin suture**				0.000	0.044 ^γ^
Silk (non-absorbable)	693 (66.6)	339 (72.6)	354 (61.8)		
Vicryl (absorbable)	265 (25.5)	91 (19.5)	174 (30.4)		
Not known	82 (7.9)	37 (7.9)	45 (7.9)		
**Suture type**				0.000	0.076 ^γ^
Interrupted	700 (67.3)	327 (70.0)	373 (65.1)		
Continuousintradermic and semisubcutaneous	260 (26.0)	96 (20.6)	174 (30.4)		
Not known	70 6.7	44 (9.4)	26 (4.5)		
**Pre-Incision Antibiotic Prophylaxis**				0.000	0.000 ^γ^
No	457 (43.9)	451 (96.6)	6 (1.0)		
Yes	572 (55.0)	10 (2.1)	562 (98.1)		
Not Known	11 (1.1)	6 (1.3)	5 (0.9)		
**Timing of Pre-Incision Antibiotic Prophylaxis**				0.017	0.011 ^γ^
Appropriate	162 (28.3)	5 (50)	157 (27.9)		
Not appropriate	372 (65)	2 (20)	370 (65.8)		
Not known	38 (6.7)	3 (30)	35 (6.3)		
**Post-Incysion Antibiotic administration**				0.000	0.000 ^γ^
No	406 (39.0)	4 (0.9)	402 (70.2)		
Yes	627 (60.3)	459 (98.2)	168 (29.3)		
Not Known	7 (0.7)	4 (0.9)	3 (0.5)		
**Day of first visit, mean, range (±SD)**	8.5, 1–38 (3.4)	8.5, 2–37 (2.5)	8.4, 1–38 (4.0)	0.701	
**Wound infection**				0.000	0.001 ^β^
Yes	320 (30.8)	225 (48.2)	95 (16.6)		
No	720 (69.2)	242 (51.8)	478 (83.4)		
**Wound infection at first visit**				0.037	0.001 ^γ^
Yes	224 (21.5)	166 (73.8)	58 (61.1)		
No	742 (71.3)	57 (25.3)	37 (38.9)		
Not known	2 (0.2)	2 (0.9)	0		
**Type of wound infection**				0.000	0.045 ^γ^
Superficial	218 (21.0)	138 (61.3)	80 (84.2)		
Deep/involving organ and/or spaces	85 (8.2)	74 (32.9)	11 (11.6)		
Not known	17 (1.6)	13 (5.8)	4 (4.2)		

*CS* caesarean section, *SSI* Surgical Site Infections; *Bonferroni adjusted *p*-value; ^β^ α = 0.012; ^γ^α = 0.008

In the POST-Int study, a greater CS number was performed by experienced surgeons (adj. *p* = 0.052) and pre-incision antibiotic prophylaxis was administered in more cases (adj. *p* < 0.001). The timing of prophylaxis was reported to be adequate only in 28% of cases in the POST-Int group, but this did not seem to affect SSI prevalence. On the other hand, post-operative antibiotic administration was significantly lower in the POST-Int group (adj. *p* < 0.001). Skin disinfection was performed using povidone/iodine more often in POST-Int operations (adj. *p* = 0.001). In the POST-Int group *Pfannenstiel* incision, absorbable and continuous intradermic/semi-subcutaneous suture were more often adopted compared to PRE-Int group, but this finding was not significant after Bonferroni correction. The mean number of days of hospitalization was similar in both groups (approximately 2 days).

### Rate and determinants of SSIs in the PRE-Int and POST-Int populations

Overall, 320 (30.8%) SSIs were reported in all enrolled women, 225 (48.2%) in the PRE-Int group and 95 (16.6%) in the POST-Int group (adj. *p* = 0.001) (Table 1). SSI diagnosis at first visit (approximately 8 days after CS) was higher in the PRE-Int group (adj. *p* = 0.001). The analysis of CS-SSI determinant factors is showed in table 2 (Table 2).

**Table 2:** Analysis of risk factors for SSI in patients undergoing CS in the PRE-Int and POST-Int groups and Overall

	Overall (*n* = 1040)			PRE-Int (*n* = 467)			POST-Int (*n* = 573)		
	SSI (*n* = 320)	No SSI (*n* = 720)	*p*-value	SSI (*n* = 225)	No SSI (*n* = 242)	*p*-value	SSI (*n* = 95)	No SSI (*n* = 478)	*p*-value
**Age, years**			0.403			0.561			0.009
< 20	58 (35.2)	107 (64.8)		33 (43.4)	43 (56.6)		25 (28.1)	64 (71.9)	
21–34	220 (29.9)	516 (70.1)		164 (48.4)	175 (51.6)		56 (14.1)	341 (85.9)	
> 34	39 (29.8)	92 (70.2)		27 (52.9)	24 (47.1)		12 (15.0)	68 (85.0)	
Not known				1 (100)	0		2 (28.6)	5 (71.4)	
**BMI, median (range)**	27.8 (14–46)	26.9 (16–66)	0.031	27.7 (19–46)	26.7 (18–45)	0.060	27.8 (14–41)	27 (16–66)	0.224
**Education**			0.327			0.518			0.621
No	46 (35.1)	85 (64.9)		34 (51.5)	32 (48.5)		12 (18.5)	53 (81.5)	
Standard 7^a^	165 (31.2)	364 (68.8)		112 (46.5)	129 (53.5)		53 (18.4)	235 (81.6)	
Form I-VI^b^	88 (30.4)	201 (69.6)		68 (51.5)	64 (48.5)		20 (12.7)	137 (87.3)	
College/University	16 (21.1)	60 (78.9)		7 (33.3)	14 (66.7)		9 (16.4)	46 (83.6)	
Not known	5 (33.3)	10 (66.7)		4 (57.1)	3 (42.9)		1 (12.5)	7 (87.5)	
**HIV+**			0.097			0.462			0.104
Yes	16 (44.4)	20 (55.6)		10 (58.8)	7 (41.2)		6 (31.6)	13 (68.4)	
No	295 (30.4)	676 (69.6)		211 (48.2)	227 (51.8)		84 (15.8)	449 (84.2)	
Not known	9 (27.3)	24 (72.7)		4 (33.3)	8 (66.7)		5 (23.8)	16 (76.2)	
**CS type**			0.607			0.469			0.338
Elective	21 (27.6)	55 (72.4)		18 (42.9)	24 (57.1)		3 (8.8)	31 (91.2)	
Emergency	299 (21.0)	665 (69.0)		207 (48.7)	218 (51.3)		92 (17.1)	447 (82.9)	
**Indication for CS**			0.801			0.558			0.084
Previously known^c^	133 (30.8)	299 (69.2)		104 (49.5)	106 (50.5)		29 (13.1)	193 (86.9)	
Not previously known	187 (30.8)	420 (69.2)		121 (47.3)	135 (52.7)		66 (18.8)	285 (81.2)	
**Operator grade**			0.006			0.038			1.000
Junior	229 (33.4)	456 (66.6)		172 (51.0)	165 (49.0)		57 (16.4)	291 (83.6)	
Senior	87 (25.2)	258 (74.8)		51 (40.2)	76 (59.8)		36 (16.5)	182 (83.5)	
**Skin disinfection**			< 0.001			0.410			1.000
Dettol/Ethanol	48 (50.0)	48 (50.0)		47 (52.2)	43 (47.8)		1 (16.7)	5 (83.3)	
Povidone-Iodine	265 (28.7)	658 (71.3)		172 (47.0)	194 (53.0)		93 (16.7)	464 (83.3)	
**Type of incision**			< 0.001			< 0.001			< 0.001
Midline Vertical	280 (38.0)	457 (62.0)		198 (58.1)	143 (41.9)		82 (20.7)	152 (92.1)	
*Pfannenstiel*	37 (15.0)	210 (85.0)		24 (29.3)	58 (70.7)		13 (7.9)	314 (79.3)	
**Skin suture**			< 0.001			0.370			0.006
Silk	237 (34.2)	456 (65.8)		167 (49.3)	172 (50.7)		70 (19.8)	284 (80.2)	
Vicryl (absorbable)	58 (21.9)	207 (78.1)		40 (44.0)	51 (56.0)		18 (10.3)	156 (89.7)	
**Suture type**			< 0.001			< 0.001			< 0.001
Interrupted	268 (38.3)	432 (61.7)		190 (58.1)	137 (41.9)		78 (20.9)	295 (79.1)	
Continuous intradermic and semisubcutaneous	45 (16.7)	225 (83.3)		31 (32.3)	65 (67.7)		14 (8.0)	160 (92.0)	
**Pre-Incision Prophylaxis**			< 0.001			1.000			1.000
No	216 (47.3)	241 (52.7)		215 (47.7)	236 (52.3)		1 (16.7)	5 (83.3)	
Yes	97 (17.0)	475 (83.0)		5 (50.0)	5 (50.0)		92 (16.4)	470 (83.6)	
**Timing of pre-Incision Prophylaxis**			0.548			0.428			0.307
Adequate	25 (27.2)	137 (31)		3 (100)	2 (50)		22 (24.7)	135 (30.8)	
Not adequate	67 (72.8)	305 (69)		0	2 (50)		67 (75.3)	303 (69.2)	
**Post-CS ATB administration**			< 0.001			1.000			0.220
No	64 (15.8)	342 (84.2)		2 (50.0)	2 (50.0)		62 (15.4)	340 (84.6)	
Yes	253 (40.4)	374 (59.6)		220 (47.9)	239 (52.1)		33 (19.6)	135 (80.4)	

^a^Primary education; ^b^Secondary education; ^c^cephalopelvic disproportion, bad obstetric history, previous CS

In the overall and per-group analysis the risk of CS SSI was lower in case of *Pfannenstiel* incision (overall: OR 0.288; 95%CI 0.197–0.420; *p* < 0.001) and of continuous intradermic/semi-subcutaneous suture (overall: OR 0.322; 95%CI 0.226–0.460; *p* < 0.001). In the PRE-Int group only, a higher experience of the surgeon was significantly associated with a lower CS-SSI risk (OR 0.644; 95%CI 0.425–0.974; *p* = 0.038). In the POST-Int group, younger age of the patient was significantly associated with higher risk of SSI (OR 2.379; 95%CI 1.384–4.089; *p* = 0.001), whereas the use of absorbable stitches was found to be protective (OR 0.468; 95%CI 0.269–0.814; *p* = 0.006). This finding was confirmed when pooled data from the two studies were combined (OR 0.539; 95%CI 0.387–0.750; *p* < 0.001).

In the PRE-Int and POST-Int studies no significant SSI differences with respect of skin disinfection and body mass index (BMI) were reported. Nevertheless, in all enrolled population the use of povidone-iodine and a low BMI were significantly associated with a lower SSI risk (*p* = 0.031 and *p* < 0.001, respectively).

Multivariate analysis showed an independent association between SSI and lack of pre-incision antibiotic prophylaxis (OR 3.588; 95%CI 1.922–6.696; *p* < 0.001) and skin disinfection performed with Dettol or Ethanol (OR 2.396; 95%CI 1.000–5.738; *p* = 0.050). Conversely, the utilization of absorbable suture for skin closure was an independent protective factor (OR 0.522; 95%CI 0.280–0.970; *p* = 0.040), together with a normal BMI (18,5-24,9) (OR 0.632; 95%CI 0.403–0.990; *p* = 0.045).

### Microbiological characteristics

Microbiological characteristics are displayed in Table 3. The rate of microbiologically-confirmed SSIs was considerably higher in the POST-Int study compared with the previous survey (OR 2.534; 95% CI 1.435–4.475; *p* = 0.001). The prevalence of SSIs caused by gram-positive bacteria significantly decreased (OR 0.263; 95% CI 0.126–0.548; *p* < 0.001), and the MRSA prevalence rate dropped down from 79 to 21.4% (OR 0.072; 95% CI 0.016–0.314; *p* < 0.001). All MRSA isolates detected in the second survey had an inducible resistance to clindamycin (data not available in the first survey). Overall, 16 (43.2%), 5 (13.5%) and 2 (5.4%) MRSA isolates were susceptible to tetracycline, chloramphenicol and cotrimoxazole, respectively (data not shown in the table). *Enterococcus spp* was not identified in the PRE-Int study while in the POST-Int accounted for 16.1% of the pathogens isolated. Eighty per cent of the *Enterococcus spp* were resistant to ampicillin. The prevalence of SSIs due to gram-negative (including *Klebsiella spp* and *Pseudomonas spp*) significantly increased in the POST-Int study (OR 3.800; 95% CI 1.822–7.926; *p* < 0.001). Overall, more than half gram-negative had a phenotypic profile consistent with MDROs. Finally, no resistances to vancomicin and meropenem were observed.

**Table 3:** Overall and per-groups analysis of microbiological characteristics

	Overall SSI (*n* = 320)	PRE-Int SSI (*n* = 225)	POST-Int SSI (*n* = 95)	*p*-value
**Swab Performed, n (col %)**				**0.887**
Yes	280 (87.5)	197 (87.5)	83 (87.4)	
No	40 (12.5)	28 (12.5)	12 (12.6)	
**Swab result, n (col %)**	***n*** **= 280**	***n*** **= 197**	***n*** **= 83**	**0.001**
No bacterial growth	112 (40)	91 (45.2)	21 (25.3)	
Bacterial Growth	168 (60)	106 (53.8)	62 (74.7)	
**Isolated pathogen, n (col %)**	***n*** **= 168**	***n*** **= 106**	***n*** **= 62**	**< 0.001**
Gram-positive	127 (75.6)	90 (84.9)	37 (59.7)	
Gram-negative	41 (24.4)	16 (15.1)	25 (40.3)	
**Gram positive, n (col %)**	***n*** **= 127**	***n*** **= 90**	***n*** **= 37**	
*Staphylococcus aureus*	57 (44.9)	43 (40.6)	14 (37.9)	
*MRSA*	*37 (64.9)*	*34 (79.0)*	*3 (21.4)*	
*Coagulase-negative Staphylococcus species*	57 (44.9)	47 (44.4)	10 (27.0)	
*Streptococcus A*	3 (2.4)	0	3 (8.1)	
*Enterococcus spp.*	10 (7.8)	0	10 (27.0)	
**Gram negative, n (%)**	***n*** **= 41**	**n = 16**	***n*** **= 25**	
*Escherichia Coli*	4 (9.8)	1 (6.3)	3 (12.0)	
*MDROs*	*2 (50.0)*	*1 (100)*	*1 (33.4)*	
*Proteus mirabilis*	4 (9.8)	3 (18.7)	1 (4.0)	
*MDROs*	*1 (25.0)*	*1 (33.4)*	*0*	
*Klebsiella spp.*	11 (26.8)	4 (25.0)	7 (28.0)	
*MDR0s*	*7 (63.6)*	*4 (100)*	*3 (42.8)*	
*Pseudomonas aeruginosa*	6 (14.6)	1 (6.3)	5 (20.0)	
*MDROs*	*4 (66.7)*	*1 (100)*	*3 (60.0)*	
*Other GRAM-negative*	16 (39.0)	7 (43.7)	9 (36.0)	
*MDROs*	*7 (43.7)*	*2 (28.6)*	*5 (55.6)*	
**Total MRSA, n (isolated pathogens %)**	**37 (22)**	**34 (32.0)**	**3 (4.8)**	**< 0.001**
**Total gram negative MDROs, n (isolated pathogens %)**	**22 (13)**	**9 (8.5)**	**12 (19.3)**	**0.052**

*MRSA Methicillin-Resistant Staphylococcus aureus*, *MDROs Multidrug Resistant Organisms*

## Discussion

A substantial decrease in SSI rates was observed after the introduction of a combined IPC with AMS joint program.

### Impact of IPC and AMS intervention on CS SSI incidence

This multimodal approach led to a percentage reduction in the CS SSIs rates by more than 65%. Furthermore, less SSIs were detected at first visit (8 days after the CS) in the POST-Int group, possibly suggesting that fewer infections were acquired in the theatre room and during inpatient stay [17]. Strengthening the chain supply for antibiotics and skin disinfectant was a priority. In the POST-Int study almost every CS was preceded by an antibiotic prophylaxis, and the skin disinfection was performed following a standardized procedure in the vast majority of cases. The AMS intervention led to a better appropriateness of antibiotic prescription, as documented also by other studies in LMICs [18]. Post-CS antimicrobial prescription was limited to cases requiring a treatment. Administering multiple doses of post-operative antibiotics was not associated with a better outcome compared with pre-operative single dose prophylaxis, as also reported by other authors [19, 20]. Furthermore, by administering a pre-operative prophylaxis instead of a 3 days intravenous post-operative course, we estimate that hospital total cost savings have been around €1500 during the Post-Int study. Moreover, a reduction of post-discharge antibiotics prescription by approximately 70% was achieved. This led to a personal saving of around €3/patient in a country where 28% of the population is estimated to live under the national basic needs poverty line (approximately €14/month per adult) [21]. These findings suggest that AMS programmes are highly recommended in LMICs to improve the prescription attitude and reduce the burden of health-care associated costs.

The study was not designed to assess the impact of each prevention measure on the occurrence of SSIs. A study with a more robust design, such as an interrupted time series analysis or a controlled randomized clinical trial would be useful to better address this issue [22, 23].

### Risk factors for post-CS before and after the intervention

Formal education and on-job training of the health-care personnel were essential components of this intervention. In particular, after sharing literature evidences, including reviews [24–27] and guidelines, we observed improvements in the operating room discipline and a more rigorous compliance to SOPs and reporting tools. The supervision of younger doctors was encouraged according with literature [12, 25, 28]. Therefore, in the POST-Int study, a greater number of CS were performed by more experienced doctors. Higher rates of *Pfannenstiel* incision, povidone-iodine for skin disinfection and absorbable continuous/semi-subcutaneous sutures for skin closure were also observed in the POST-Int. Absorbable suture and disinfection with povidone-iodine were associated with lower risk of SSIs at multivariate analysis. Literature-based evidences suggest that subcutaneous tissue closure is associated with fewer wound complications [29], but the outcome may be different according with the type of CS incision [30]. Further well-designed trials at low risk of bias are needed to provide non-conflicting results on the best type of suturing.

Data retrieved did not allow for the evaluation of other factors that may have an impact on the risk of developing SSI post-CS, including diabetes, chorioamnionitis and hypertension. This must be considered a setting-related limitation of the present study.

### Changes in the characteristics of the isolates after the intervention

A greater proportion of microbiologically-confirmed SSIs in the POST-Int study was reported, possibly related to a better collection and processing of the samples. Also the laboratory diagnostic capability seemed to be improved, as suggested by the detection of *Enterococcus spp,* which was not identified in the PRE-Int study. In 2016 the DRRH Laboratory has been audited per the WHO AFRO Stepwise Laboratory Quality Improvement Process Towards Accreditation (SLIPTA) Checklist and has met the requirement for “three star” recognition level.

The significant reduction of gram-positive strains isolated and the lower MRSA prevalence in the second survey are noteworthy. This is likely a consequence of the AMS education and on-job training which dramatically affected the health care workers’ attitude and behaviour towards antibiotic prescription and IPC [31]. By avoiding unnecessary use of ceftriaxone in the post-operative period, the intervention may have contributed to the reduction in MRSA rates in the second survey. The choice of Ampicillin for prophylaxis depended on the unavailability of cefazolin, that is more widely used for surgical prophylaxis and more effective against *Staphylococcus aureus* [32]. Probably, this is the reason why we observed a similar rate in MSSA before and after the intervention. On the other hand, considering the unavailability of second line antibiotics in the majority of health facilities in Tanzania, the high rate of MDROs - including gram-negative bacteria – detected in this study is a serious concern. The lack of detailed data on MDROs prevalence all over the country highlights the need for building an efficient surveillance system in order to guide continuous targeted interventions.

## Conclusions

The intervention implemented within this study was a multidisciplinary collaborative strategy involving, among the others, the hospital management, the pharmacist, the microbiologist and the dedicated nurse. This AMS Multidisciplinary Team worked to improve the SSI prevention, surgical procedures and laboratory capability and cooperated to ensure a better management of wound infections. The participation of different health care figures could be key to obtain an efficient hospital based surveillance system, able to detect the gap, prioritize interventions and inform national and international institutions on the magnitude of AMR [4].

This multimodal approach demonstrated to be effective in a resource-constrained setting and highlighted the need to improve IPC and AMR education of hospital staff [9]. Improved SSI and AMR surveillance is warranted to better understand the occurrence of SSIs and their microbiological characteristics. Further IPC-AMS joint programmes with robust study designs should be encouraged and scaled up in LMICs.

## Data Availability

The datasets used and/or analysed during the current study are available from the corresponding author on reasonable request.

## Abbreviations

AMR: Antimicrobial Resistance
AMS: Antimicrobial Stewardship
BMI: Body Mass Index
CDC: Center for Diseases Control
CS: Caesarean Section
DRRH: Dodoma Regional Referral Hospital
HAI: Health-care Associated Infection
ID: Infectious Diseases
IPC: Infection Prevention and Control
LMIC: Low and Middle Income Country
MDRO: Multidrug Resistant Organisms
MRSA: Methicillin-Resistant *Staphylococcus aureus*
OGD: Obstetrics & Gynaecology Department
POST-Int: Post-intervention survey
PRE- Int: Pre-intervention survey
RCID: Resource Centre for Infectious Diseases
SOP: Standard Operating Procedure
SSIs: Surgical site infections
WHO: World Health Organization
